# Mortality Implications of Increased Active Mobility for a Proposed Regional Transportation Emission Cap-and-Invest Program

**DOI:** 10.1007/s11524-020-00510-1

**Published:** 2021-01-20

**Authors:** Matthew Raifman, Kathy Fallon Lambert, Jonathan I. Levy, Patrick L. Kinney

**Affiliations:** 1 Department of Environmental Health, Boston University, Boston, MA USA; 2 Harvard T.H. Chan School of Public Health, Boston, MA USA

**Keywords:** Physical activity, Transportation, Active transport, Bicycling, Walking, Mortality

## Abstract

The transportation sector is now the primary contributor to greenhouse gas emissions in the USA. The Transportation Climate Initiative (TCI), a partnership of 12 states and the District of Columbia currently under development, would implement a cap-and-invest program to reduce transportation sector emissions across the Northeast and Mid-Atlantic region, including substantial investment in cycling and pedestrian infrastructure. Using outputs from an investment scenario model and the World Health Organization Health Economic Assessment Tool methodology, we estimate the mortality implications of increased active mobility and their monetized value for three different investment allocation scenarios considered by TCI policymakers. We conduct these analyses for all 378 counties in the TCI region. We find that even for the scenario with the smallest investment in active mobility, when it is fully implemented, TCI would result in hundreds of fewer deaths per year across the region, with monetized benefits in the billions of dollars annually. Under all scenarios considered, the monetized benefits from deaths avoided substantially exceed the direct infrastructure costs of investment. We conclude that investing proceeds in active mobility infrastructure is a cost-effective way of reducing mortality, especially in urban areas, providing a strong motivation for investment in modernization of the transportation system and further evidence of the health co-benefits of climate action.

**Supplementary Information:**

The online version contains supplementary material available at 10.1007/s11524-020-00510-1.

## Introduction

In the USA, the transportation sector produces the largest share (28.2%) of greenhouse gas (GHG) emissions [1]. In the Northeast and Mid-Atlantic states, transportation accounts for an even greater share (34.5%) of CO_2_ equivalent emissions [2]. Building upon existing programs (e.g., the Regional Greenhouse Gas Initiative) [3], 12 states and the District of Columbia are currently in discussions to create a program called the Transportation Climate Initiative (TCI). The TCI is a regional cap-and-invest program that is intended to limit GHG emissions from the transportation sector and further reduce emissions through investment of proceeds in sustainable transportation initiatives.

Actions to reduce GHG emissions from the transportation sector present an opportunity to both mitigate climate change and provide near-term health co-benefits, which could be important motivation for policymakers and stakeholders. Strategies to reduce transportation GHG emissions will reduce emissions of other air pollutants shown to be harmful to health [4, 5]. In addition, TCI is expected to have significant impact on mobility choices across the region both by internalizing the negative externalities of personal occupancy vehicle travel through a price signal and by improving alternative transportation options through investment in sustainable transportation. One of the most prominent opportunities to simultaneously reduce transportation emissions and improve health, particularly in urban areas, is investment in active mobility.

Active mobility, defined as transportation modes that are human-powered (primarily walking and cycling), is associated with improved health outcomes, including reduced risk of all-cause mortality [6], lower incidence of cardiovascular disease [7] and diabetes [8], and improved mental health [9]. Further, research suggests that investing in cycling [10] and pedestrian [11] infrastructure can increase the likelihood of active mobility trips. By investing some of the proceeds from the TCI program in active mobility infrastructure, there is the potential to shift residents to active modes that will improve health and create a virtuous cycle of further reductions in GHG emissions.

In this study, we quantified the potential mortality impacts of increased active mobility under illustrative TCI cap-and-invest scenarios in the 13 jurisdictions. As inputs, we used estimates of the change in personal miles traveled (PMT) cycling and walking under three different investment scenarios developed by state policymakers in the TCI region. We implemented the World Health Organization (WHO) Health Economic Assessment Tool (HEAT) methodology to estimate the mortality implications from increased active mobility for all 378 counties in the region, taking into account deaths avoided from increased physical activity and additional traffic fatalities incurred from increased PMT walking and cycling. Finally, we present results for net deaths avoided and monetized benefits by county, highlighting the potential health benefits of the illustrative TCI scenarios particularly within urban areas.

## Methods

### Greenhouse Gas Reductions and Investment Policy Scenarios

As currently envisioned in the draft TCI Memorandum of Understanding [12], fuel suppliers of on-road diesel fuel and finished motor gasoline will be required to hold allowances for each ton of GHG emissions based on the carbon content of the fuel they sell. As with other emission cap programs, the number of allowances will be determined based on a target emission level (or “cap”) that can be ratcheted down to reduce emissions over time. Fuel suppliers would need to purchase allowances on the market each year, which would influence the price of gasoline and generate revenue for states to invest. Each state that is a signatory to the final TCI program would receive a share of the allowance auction proceeds and maintain discretion to invest proceeds as desired, with the overall understanding that states will prioritize sustainable transportation and equity [12].

TCI would generate revenue by implementing a price on transportation CO_2_ emissions, of which a portion would be invested in cycling and pedestrian infrastructure improvements. State policymakers have considered a number of different scenarios, including various caps and allocations of proceeds. Here, we evaluate the mortality implications of changes in active mobility from three illustrative investment scenarios (“A,” “B,” and “C”) under a 25% GHG emission cap in 2032. Each investment scenario has a different mix across six buckets, including fleet electrification and alternative fuel incentives, vehicle travel reduction, road infrastructure improvements, urban transit expansion, transit vehicle investment, and indirect non-GHG reducing investment (see Table 1) [13]. Investment in pedestrian and cycling infrastructure is found within the travel reduction bucket. While the emission cap is the same across all three scenarios considered here, total proceed to be allocated is not, as the total revenue generated by a cap is the product of the interaction between the investment allocation and petroleum fuel demand (a detailed breakdown of investment allocation under each scenario and the impacts of six additional emission cap-and-invest scenarios can be found in the appendix and demonstrate that benefits scale roughly linearly across the various investment levels).

**Table 1:** TCI investment scenarios for a 25% cap on regional GHG emissions with active mobility investments broken down; annual investment presented is averaged over the 2022–2032 period. See Appendix Table 4 for a complete breakdown of all investment buckets

Investment bucket	Scenario A		Scenario B		Scenario C	
	Share of total	Avg. annual investment (millions)	Share of total	Avg. annual investment (millions)	Share of total	Avg. annual investment (millions)
Fleet EV/Alt. fuel Incentives	25.9%	$2000	52.7%	$3300	81.3%	$3700
Vehicle travel reduction	16.2%	$1200	13.9%	$870	10.5%	$480
Bicycle investment	4.2%	$320	5.1%	$320	6.0%	$280
Pedestrian investment	4.2%	$320	3.0%	$190	0.0%	$0.0
Other	7.8%	$560	5.8%	$360	4.5%	$200
Road infrastructure	7.0%	$530	7.6%	$480	0.8%	$36
Urban transit expansion	24.2%	$1800	12.4%	$780	0.0%	$0.0
Transit vehicle investment	10.0%	$760	5.0%	$310	0.0%	$0.0
Indirect non-GHG reducing	16.7%	$1300	8.3%	$520	7.4%	$340
Total	100.0%	$7600	100.0%	$6300	100.0%	$4600

Scenario A represents a diversified investment portfolio where each of the six buckets receives a balanced allocation of the revenue. In this scenario, walking and cycling infrastructure both receive an average annual allocation of $320 million. Scenario C prioritizes investments that achieve the greatest reductions in GHG emissions per dollar invested. Finally, scenario B is a middle ground mix between the other two scenarios. In the TCI investment model (described below), pedestrian infrastructure is less cost-effective at reducing GHG emissions compared to electrifying public transit, cycling infrastructure, and several other investment options. As a result, pedestrian infrastructure is deprioritized in scenario B compared to scenario A, and eliminated altogether from scenario C.

### Estimating the Impact of TCI Investment on Active Mobility

**Fig. 1:**
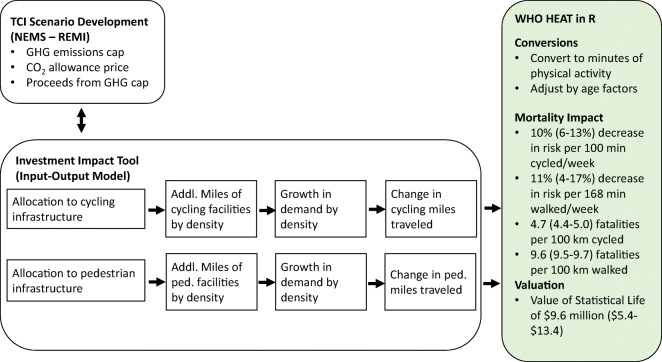
Schematic of multi-model study design

To compare illustrative scenarios under consideration by the policymakers, the TCI implemented an input-output tool [14] (“Investment Strategy Tool”) to model how investment of TCI proceeds in various sustainable transportation strategies might shift vehicle and personal miles traveled. In the case of active mobility, the model estimated the change in PMT walking and cycling from investment in cycling and pedestrian infrastructure. We use the output from this tool (county-level change in PMT) as the input for our mortality assessment from estimated increased activity (see Fig. 1). Below, we briefly describe the modeling assumptions for the Investment Strategy Tool pertaining to active mobility investment. (For a more comprehensive description of the Tool methodology, please see the documentation on the Transportation Climate Initiative website [15].)

#### Investment of TCI Proceeds in Cycling Infrastructure

The investment tool is based on the assumption that investment in cycling infrastructure (specifically four facility types: bike lanes, separated bike lanes, shared-used paths, and bike boulevards) will incent mode shift to cycling and increase the number of personal miles cycled across the region. The estimated increase in bicycle PMT from a dollar invested in cycling infrastructure is a function of both (1) the cost per facility mile and (2) the change in miles traveled from the construction of a new facility mile. Both of these factors themselves are a function of county population density and facility type. Put differently, the tool assumes that a mile of bike lane differs in cost ($25,000) from a mile of grade-separated protected bike lane ($500,000), and the impact of a mile of newly constructed bike lane on PMT per capita is assumed to be different for an urban county compared to a rural one. The increase in bicycles’ miles traveled per new facility mile is based on various studies in US cities examining the relationship between bicycle infrastructure and bicycling [10, 16, 17]. Maximum network density assumptions (quarter-mile spacing for bike lanes, one-mile spacing for separated paths) limit the extent of the network. Funding is initially allocated across population density categories in proportion to the population in each category, but is capped once the network is built out in a county density type, which happens in the more densely populated counties.

#### Investment of TCI Proceeds in Pedestrian Infrastructure

The pedestrian investment model is based on a “Complete Streets” framework [18], which assumes that investment in sidewalks, traffic calming, and other pedestrian-focused strategies will make walking safer and more attractive, leading to increased PMT walking. As with the cycling investment model, it is assumed that the construction of Complete Streets projects will increase walking trips as a function of county population density. Pedestrian infrastructure is assumed to have greater impact on the number of walk trips in urban areas compared to rural areas; however, the capital cost of an additional facility mile of Complete Streets projects (incremental to the cost of other roadway improvements) is assumed to be more expensive in urban core areas ($900,000) compared to rural areas ($250,000). The TCI tool does not implement a maximum build-out for pedestrian infrastructure, but it does take into account existing pedestrian mode share.

In addition, separate investment in public transit is assumed to generate an increase in walk PMT due to the multi-modality of transit commuting. The investment model applies the blanket assumption that all new transit trips from TCI investment will include a ¼-mile walk on each end. The tool assumes that cycling and walking investments are made independently and do not affect change in PMT of each other. In practice, it is conceivable that TCI investment in shared use paths (a type of cycling investment) might increase pedestrian PMT as well as cycling PMT, making this a conservative assumption regarding pedestrian PMT.

#### Time Horizon

For both cycling and pedestrian infrastructure investment, a 1-year time lag is assumed to occur between investment and completion of construction. While the TCI initiative is expected to be implemented in 2022, with revenue invested annually thereafter in new cycling and pedestrian infrastructure, for our analysis, we assume full build-out of TCI-funded cycling and pedestrian infrastructure will occur in 2032. We estimate annual mortality benefits starting in 2032 and going forward, ignoring partial benefits occurring during the build-out period.

### Estimating the Impacts of Additional Active Mobility on Mortality

We developed an R [19] version of the WHO HEAT [20] tool to estimate how the change in PMT in each investment scenario would impact mortality incidence at the county level. We estimated both deaths avoided from increased physical activity and additional traffic fatalities incurred from increased cycling and biking. We used the concentration-response functions for deaths avoided from increased walking and cycling activity that were agreed upon by the WHO after reviewing the literature (RR: 0.89; 95% CI: 0.83, 0.96 for walking and RR: 0.90; 95% CI: 0.87–0.94 for cycling per 11.25 metabolic equivalents (MET) hours, a measure of physical activity exertion, per week from a meta-analysis of the existing literature) [6]. We applied the traffic fatalities per 100 million kilometers cycled (4.7; 95% CI: 4.4–5.0) and walked (9.6; 95% CI: 9.5–9.7) found in Buehler and Pucher (2017) [21] to estimate the increase in traffic fatalities from additional PMT under each scenario. Finally, we employed travel speed assumptions and minutes per MET-hour of activity used by the WHO to convert miles traveled to minutes of activity for use in the HEAT functions. Pulling these assumptions together, we related a change in minutes of activity to a change in net deaths through the following relationships at the county level:

$$ \mathrm{deaths}\ \mathrm{avoided}=\left(1-\mathrm{RR}\right)\ast \frac{\mathrm{change}\ \mathrm{in}\ \mathrm{minutes}\ \mathrm{of}\ \mathrm{activity}}{\mathrm{reference}\ \mathrm{activity}\ \mathrm{value}}\ast \mathrm{baseline}\ \mathrm{mortality}\ \mathrm{rate} $$

$$ \mathrm{traffic}\ \mathrm{fatalities}=\left(\frac{\mathrm{change}\ \mathrm{in}\ \mathrm{PMT}}{100\ \mathrm{million}}\right)\ast \mathrm{fatality}\ \mathrm{rate}\ \mathrm{per}\ 100\ \mathrm{million}\ \mathrm{miles}\ \mathrm{traveled} $$

$$ \mathrm{net}\ \mathrm{deaths}\ \mathrm{avoided}=\mathrm{deaths}\ \mathrm{avoided}-\mathrm{traffic}\ \mathrm{fatalities} $$

where the change in minutes of activity is calculated assuming a cycling speed of 8.7 mph and walking speed of 3.3 mph, and the reference activity level needed to achieve the full protective effect is 100 min for cycling and 168 min per week for walking based on the MET-to-minutes conversion.

The concentration-response functions for deaths avoided from increased physical activity are specifically for adults, aged 20–64 for cycling and 20–74 for walking [20]. To be conservative, we assume that only a portion of the change in PMT would occur within these relevant age groups. We used the 2017 National Household Travel Survey [22] to estimate the share of survey-weighted bike miles (80%) and pedestrian miles (72%) traveled by each age group and applied these factors to our PMT inputs.

Mortality rate is correlated with age, race/ethnicity, income, and many chronic conditions. Most notably, mortality rate increases exponentially with age even within the 20–64 years old group. Using the HEAT methodology, given the same change in activity, areas with older populations and thus higher baseline mortality will see larger reductions in mortality from increased activity. While we incorporate differential baseline mortality rates *between* age groups, we assume homogeneity of mortality risk *within* age groups. We also assume homogeneity across the age range for the distribution of change in PMT. Data from the 2017 National Household Travel Survey suggest that the current cycling and walking rate is relatively consistent across the age groups considered, though it is not necessarily the case that current adoption by age is a good proxy for behavior change by age [22].

To estimate the monetized benefits of net deaths avoided, we applied a value of statistical life (VSL) of $9.6 million (2016 USD), with a range of ($5.4 million–$13.4 million) and a uniform distribution, based on the U.S. DOT guidance [23]. We performed a Monte Carlo analysis to construct confidence intervals that combine uncertainty for the estimates of physical activity mortality impacts, traffic fatalities, and monetized benefits.

### Data Sources

To estimate the change in personal miles traveled for both walking and cycling, we used the county-level outputs from the investment tool for each of the proposed scenarios. For the county-level baseline mortality rate, we used all-cause mortality from CDC WONDER [24] averaged across the 1999–2016 period for the 20–64 age group (for cycling) and 20–74 age group (for walking), and population data from the 2019 U.S. Census Bureau’s Population Estimates Program [25].

## Results

### Change in Active Mobility by Investment Scenario

We estimated the change in activity that would result from TCI investment in cycling and walking infrastructure for the three investment scenarios: “A,” “B,” and “C” under a 25% GHG emission reduction cap for on-road transportation.

**Fig. 2:**
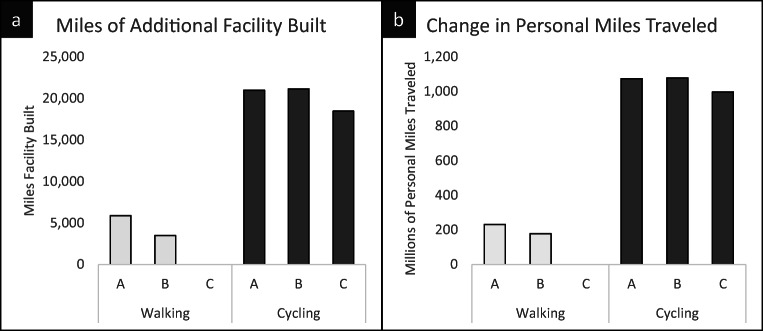
Miles of active mobility infrastructure built with TCI investment (**a**) and resulting change in active mobility (**b**) for walking and cycling under the three scenarios starting after build-out (2032)

Cycling infrastructure improvements would receive in the range of $280–$320 million per year over the 10-year investment period (2022–2032), which would result in an estimated 18,000–22,000 miles of additional cycling infrastructure across the region (see Fig. 2). We estimate that this increase in cycling infrastructure would generate around 1 billion additional cycling PMT per annum starting in 2032 after full build-out.

Investment in pedestrian infrastructure has more variation across the scenarios. In scenario “A,” TCI investment is estimated to produce 6000 miles of pedestrian facilities in the region and an estimated 230 million additional PMT per year. Scenario “B” would produce around 3500 miles of Complete Streets leading to 180 million miles of additional pedestrian PMT per year. Scenario “C” includes no investment in pedestrian infrastructure.

### Impact of Investment Scenarios on Mortality across TCI Region

We found that regardless of the investment scenario, hundreds of deaths could be avoided annually from TCI investment in active mobility infrastructure (see Table 2). Scenario “A,” the balanced portfolio approach, would net the largest number of deaths avoided, 710 (95% CI: 450, 970), corresponding with its greater investment in active mobility measures, including equal investment in cycling and walking infrastructure. The middle-of-the-road scenario “B” would result in an estimated 580 (95% CI: 380, 790) net deaths avoided per year, and the maximum GHG reduction scenario (“C”) is estimated to generate 390 (95% CI: 240, 550) net deaths avoided per year. Across all three scenarios, mortality disbenefits from traffic fatalities are approximately an order of magnitude smaller than the mortality benefits of increased physical activity.

**Table 2:** Annual mortality implications from increased active mobility under TCI investment scenarios starting in 2032. Totals may differ from the sum of the data presented due to rounding for two significant figures

	Scenario A		Scenario B		Scenario C	
	Est.	95% CI	Est.	95% CI	Est.	95% CI
Deaths avoided, activity (cycling)	490	(320, 660)	490	(320, 660)	450	(300, 610)
Deaths avoided, activity (walking)	320	(130, 510)	180	(74.0, 280)	0.00	0.00
Fatalities (cycling)	− 65.0	(− 61.0, − 69.0)	− 65.0	(− 61.0, − 69.0)	− 60.0	(− 56.0, − 64.0)
Fatalities (walking)	− 36.0	(− 35.0, − 36.0)	− 20.0	(− 20.0, − 20.0)	0.00	0.00
Net deaths avoided*	710	(450, 970)	580	(380, 790)	390	(240, 550)
Monetary value ($2016 millions)	$6800	($2700, $11000)	$5600	($2300, $8900)	$3800	($1500, $6100)

*These results do not include safety-in-numbers from increased active mobility volume, which may attenuate the relationship between miles traveled and traffic fatalities. Including this effect would increase net deaths avoided estimates to 770, 630, and 430 for scenarios A–C, respectively (see “Discussion”)

### Distribution of Mortality Benefits by County Population Density

We found that mortality benefits are likely to be concentrated in denser counties. For example, in the middle-of-the-road scenario “B,” 83% of net deaths avoided from TCI investment in active mobility would occur in counties with a density of 4000 persons or greater per square mile (Urban, Urban Core, or NYC; see Table 3). Denser counties would see both the largest nominal change in activity and the largest change per capita from TCI investment in active mobility. The concentration of activity impacts in denser areas extends to a concentration of net deaths avoided in urban counties. Notably, “Urban” counties (4000–10,000 persons per square mile) present the most efficient mix of population size, utilization, and facility cost, and would likely see the greatest change in both cycling and walking activity per capita aside from New York City given the assumptions made (see below). The relatively lower baseline mortality rate in “Urban” counties compared to “Urban Core” counties, however, attenuates the relationship between net deaths avoided per capita and density. We see the same pattern emerge in rural counties compared to suburban counties due to relatively higher baseline mortality rates in rural areas (see Appendix Table 15).

**Table 3:** Change in annual active mobility and net deaths by county population density per capita (p.c.) for the 25% GHG reduction cap, scenario “B” starting in 2032

County type	Pop. density per Sq Mi	Change in bike PMT (millions)	Change in walk PMT (millions)	Change in mike miles p.c.	Change in walk miles p.c.	Net deaths avoided	Net deaths avoided p.c.
NYC	72,000	220	86	160	50	130	10
Urban Core	> 10,000–72,000	250	48	31	4.8	160	2.2
Urban	4000–10,000	380	27	95	5.2	190	5.3
Suburban	500–3999	200	12	8.0	0.38	88	0.37
Rural	< 500	27	5.3	1.8	0.27	18	0.13

**Fig. 3:**
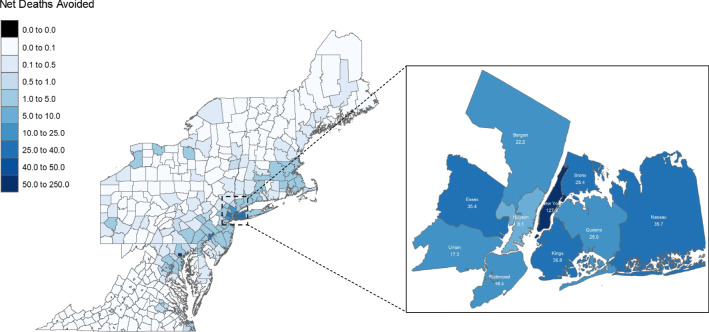
Net deaths avoided by county from increase active mobility, scenario “B” investment allocation, with pop-out for the Greater New York City area

The 11 counties in New York State and New Jersey near New York City (see Fig. 3) would account for the majority of the net deaths avoided from TCI investment in active mobility (i.e., ~ 60% in scenario “B” investment scenario) though they only account for 19% of the TCI region by population. The Greater New York area is unique in the TCI region with regard to population density, non-car mode share, county baseline mortality rates, and other factors that contribute to the concentration of active mobility benefits within its limits. Net deaths avoided are estimated to be greatest in New York County, New York (Manhattan), where an estimated increase of 220 million cycling PMT and 86 million walking PMT would result in 130 (95% CI: 78, 180) annual net deaths avoided in scenario “B.”

### Distribution of Monetized Benefits

**Fig. 4:**
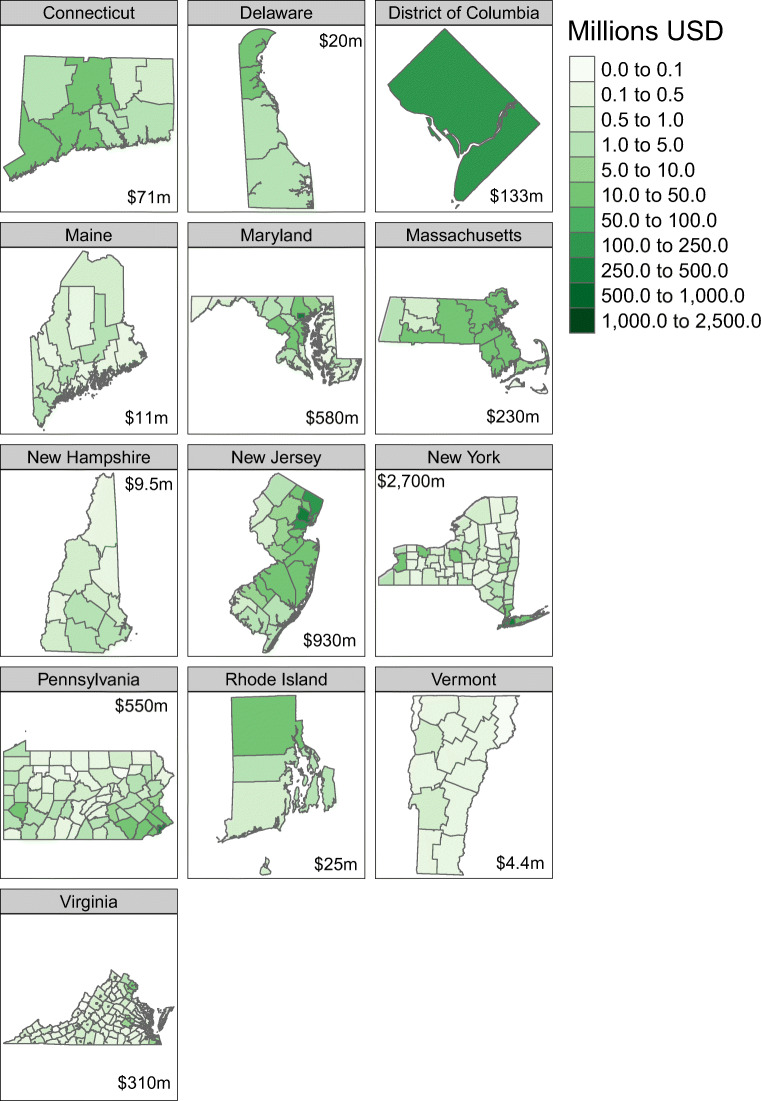
Distribution of annual monetized value of mortality benefits across states and counties for scenario “B” investment scenario starting in 2032

The potential annual monetized benefits from investment of TCI proceeds in active mobility vary by investment scenario from $3.8 billion (95% CI: $1.5 bn, $6.1 bn) in scenario “C” up to $6.8 billion (95% CI: $2.7 bn, $11 bn) in scenario “A.” Monetized benefits are also greatest in states with the highest share of population located in denser counties, namely New York and New Jersey (see Fig. 4). Again, using the middle-of-the-road scenario “B” as an example, the annual monetized benefits for New York County alone are estimated to be $1.2 billion (95% CI: $0.48 bn, $2.0 bn).

While not intended to be a complete cost-benefit analysis—we do not consider costs beyond infrastructure investment (e.g., consumer costs), or benefits beyond physical activity-based mortality (e.g., air quality and climate effects)—it is possible to compare direct infrastructure investment with estimated monetized net benefits from active mobility. In all scenarios, the annual monetized benefits from net deaths avoided would exceed infrastructure investment even after applying a 7% discount rate to account for the 10-year build-out lag from 2022 to 2032. The central estimate for monetized benefits per dollar invested in active mobility was as follows: $5.4 (“A”), $5.6 (“B”), and $6.9 (“C”). Scenario “C” interestingly has the lowest value of monetized benefits, but has the highest benefits per dollar invested. This is due to the relative cost-effectiveness of cycling investment and the elimination of pedestrian investment in that scenario. The magnitude of benefits compared to costs after discounting suggests that these results are likely to hold with all but the most extreme assumptions.

## Discussion

We estimated the mortality implications of increased active mobility under three investment scenarios that could result from the implementation of the TCI. We find that, regardless of the investment scenario implemented, TCI-induced active mobility would result in hundreds of deaths avoided per year across the region, with benefits highly concentrated in urban counties. The annual monetized benefits of net deaths avoided are estimated to be in the billions of dollars at full build-out. These results highlight that climate action on transportation directed at reducing GHG emissions can also achieve near-term health co-benefits through physical activity when cap-and-invest proceeds are invested in active mobility infrastructure.

Variation in estimated net deaths avoided across the region is driven primarily by population density, which, in this analysis, is a key input to investment allocation, infrastructure usage, and growth in active mobility, and ultimately the estimated cycling and pedestrian PMT change, which we use as the input to our health impact assessment. Our finding that the change in activity from walking and cycling investment would be concentrated in urban areas is consistent with other datasets: only 8.4% of cycling miles traveled in the USA and 13.9% of pedestrian miles traveled occurred in rural Census Block Groups in the recent 2017 National Household Travel Survey [22].

The monetized benefits per dollar invested in active mobility were greater than 1 for all three scenarios when considering exclusively infrastructure investment and active mobility-related mortality. The magnitude is inversely related to the share of investment in pedestrian infrastructure, suggesting that cycling infrastructure investment is more cost-effective. When considering investment strategies, however, cost-effectiveness should be augmented with equity considerations and investing exclusively in cycling may not be equitable. There is evidence to suggest that even after adjusting for cycling demand in urban areas, bike lanes tend to be inequitably placed in higher socioeconomic zones [26–28]. Bike lanes are most effective in dense urban areas; however, the suburbanization of poverty may result in income disparities in access to, and use of, cycling infrastructure if placement is exclusively driven by cost-effectiveness [26, 29]. To compound this, analysis of the US National Household Travel Survey has highlighted that the lowest income households (those with income less than $25,000) are ten times less likely to own a vehicle compared to those with higher incomes, and more likely to use public transit and cycling to commute to work than the nation on average [30]. Perpetuating investment in active mobility infrastructure in wealthier urban areas may not only fail to support the most marginalized among us but also underinvest in areas where there is likely to be greater utilization. These disparities in infrastructure placement could also lead to disparities in health benefits from active mobility, as disparities in baseline mortality rate exist by race and income [31]. Ultimately, the allocation of TCI proceeds is a complex process that must weigh these issues and more, including other equity considerations (e.g., rural-urban).

We have clearly underestimated the health co-benefits of TCI in this analysis by omitting air quality benefits and the direct public health benefits of GHG emission reductions. The existing literature suggests that the mortality benefits of increased activity from reducing car trips typically exceed the associated mortality benefits of improved air quality in health impact assessments. James et al. (2014) examined the impact of proposed transit fare increases in Boston and found that physical activity disbenefits from additional car trips were substantially higher (more than 10 times) than the disbenefits from air pollution and carbon emissions [32]. Grabow et al. (2012) assessed the impact of eliminating short-distance car trips in the Midwest on health through air quality and physical activity and found that the number of deaths avoided from additional activity was about 13% higher than the number of deaths avoided due to improved air quality (PM_2.5_ and O_3_) [33]. While both papers used the WHO HEAT methodology to assess activity benefits, they used different approaches to model air quality impacts, highlighting the sensitivity of results to the methods and assumptions made.

Our results may also underestimate the health benefits of investment in active mobility infrastructure because we do not estimate benefits for those under age 20 and we do not consider morbidity endpoints, including potential mental health benefits from increased activity. While physical activity has not been shown to reduce the likelihood of onset of depression and anxiety, research suggests that regular exercise (such as a daily bi-directional cycling commute) can reduce depressive symptoms and acute anxiety [34]. There is also evidence from cohort studies of a negative association between level of physical activity and risk of development of neurological disease, including dementia, Alzheimer’s, and Parkinson’s [35]. In order to remain consistent with the underlying epidemiological studies supporting the relationship between increased physical activity and mortality [6], we limited our analysis to the 20–64 age group for cycling and 20–74 age group for walking. We only had access to modeled estimates of the change in PMT for each scenario and did not have data on baseline activity levels. We thus lacked inputs necessary to estimate morbidity outcomes using physical activity modeling tools like Integrated Transport and Health Impact Modelling Tool [36].

In our analysis, we utilize one of the only exposure-based estimates available of the number of traffic fatalities per distance traveled walking and cycling in the USA [20]. This assumes that historical traffic fatality rates will apply in the future, after large-scale investment in walking and cycling infrastructure, which is a conservative assumption. There is emerging literature suggesting that as the volume of cyclists and pedestrians increases, a safety-in-numbers effect may attenuate the miles traveled-traffic fatalities relationship. As a supplemental analysis, we applied a safety-in-numbers effect estimate of 0.459 for cycling and 0.409 for walking from Elvik and Goal (2019) to our main results [37]. After incorporating the safety-in-numbers effect, we estimate that annual net deaths avoided from a 25% GHG emission cap would increase from 710 to 770 lives in scenario A, 580 to 630 in scenario B, and 390 to 430 in scenario C with total monetized benefits with safety-in-numbers of $7.4, $6.0, and $4.1 billion (USD 2016), respectively (see Appendix Table 6 for a state-by-state breakdown of net deaths avoided including the estimated impact of safety-in-numbers).

Our estimates of deaths avoided from increased active mobility vary geographically in part because of varying county baseline mortality rates. This is appropriate within the model, as it follows that the areas with the lowest mortality rate should, on average, benefit the least from additional activity given that they are relatively healthier and given the assumption of uniform relative risk across the population. As noted, baseline mortality rates can be different between counties both because of age distribution differences within the age group considered and because of differences in health conditions. The impact of overall health can be illustrated by comparing two counties that are composed entirely of urban environments, for example, Baltimore City County, Maryland (a county comprised of Baltimore City), and New York County, New York (a county comprised of Manhattan). While the cumulative change in activity is much greater for New York County than for Baltimore City, the impact of TCI investments on net deaths avoided per capita is similar for the two counties. While the two counties have similar age distributions (in 2018, median age in New York County was 2 years higher than that in Baltimore City), the baseline all-cause mortality rate is much higher in Baltimore City (921 deaths per 100,000 for the 20–70 year old group) than in New York County (366 deaths per 100,000 for the 20–70 year old group).

Within a geography, the higher risk of mortality for older ages compared to younger ages means that our mortality estimates are driven by assumed benefits to older individuals within each age group. There is limited evidence to suggest that the benefits of physical activity may actually increase with age within the 20–74 year old group [38], suggesting that this may be appropriate. We believe that this approach is acceptable for population studies like this one, but recognize that future studies could develop more refined estimates of the health impacts of specific TCI-funded projects using cause-specific mortality rates and smaller age groups for both exposure and mortality estimates.

We presented results from the three different investment scenarios under the assumption that all states will implement the same investment allocation (e.g., scenario “A”). All states, however, are not required to implement the same approach to allocating proceeds from TCI. For example, Rhode Island could opt to allocate 25% of its proceeds to active mobility and Maryland could opt to allocate none. It is also necessary to recognize the limitations of the TCI Investment Tool within the context of the fluidity of current policymaking. The input-output tool is designed to estimate the regional- or state-level impacts on miles per travel mode from investment in various low-carbon transportation strategies. Based on assumptions derived from the transportation and epidemiological literature, this approach is effective for understanding population-level average effects that might occur from the illustrative TCI investment scenarios considered during the current policy development period.

This approach, however, does not and cannot account for the impacts of specific investments and their placement (e.g., a 5-mile separated bike lane in a specific neighborhood or a sidewalk upgrade at a specific intersection), or reliably estimate the impacts of investment on specific demographic subgroups (e.g., age, urbanity, or sex), as access and utilization of active mobility infrastructure are highly sensitive to project placement. This is a limitation, as there is reason to believe that physical activity [39] benefits and traffic fatalities [40, 41] may differ by these demographic characteristics. In this analysis, we aim to walk the line between making the assumptions necessary to estimate regional effects to inform ongoing policymaking, while avoiding excessive precision that might connote a false sense of certainty given the fluidity of policymaking, resource allocation, and project planning.

We were not able to estimate uncertainties related to the transportation investment tool, such as the relationship between dollars invested and miles of new infrastructure built. Our analysis also does not incorporate the potential impacts of the novel coronavirus on transportation, which are uncertain at this time, including a possible sustained increase in work-from-home behavior, reduced transit use, and increased cycling mode share [42]. As such, confidence intervals presented here reflect uncertainty only from the relative risk associations used in the health impacts analysis and the VSL estimate. Finally, the results estimate the benefits of specific cap-and-invest scenarios and are intended to inform decision-making, but not to predict the actual benefits of the program given that proceeds may vary, and investment allocations have not yet been determined. By evaluating several different illustrative investment scenarios, we do inherently provide some consideration of the sensitivity of the mortality results to varying levels of investment; however, the true uncertainty is likely greater than that captured here.

We believe our results have the potential to inform policymaking currently underway, as states are in the process of designing the TCI program and determining how proceeds will be invested. Our results convey a subset of the health co-benefits associated with sustainable transport investment. By putting a price on GHG emissions from the transportation sector, TCI can both reduce the sector’s contribution to climate change and generate resources that can be invested to accelerate climate action and improve health. We find that investing proceeds in active mobility infrastructure is a cost-effective way of reducing mortality, especially in urban areas, providing a strong motivation for investment in modernization of the transportation system.

## Supplementary Information

ESM 1 (DOCX 63.0 kb)
