# Supplementary material for: Mortality Implications of Increased Active Mobility for a Proposed Regional Transportation Emission Cap-and-Invest Program
Source: J Urban Health. 2021 Jan 20;98(3):315–27. doi: 10.1007/s11524-020-00510-1 (PMC7816754; doi:10.1007/s11524-020-00510-1)
Supplement: Supplementary file 1 — (DOCX 63.0 kb) [file 11524_2020_510_MOESM1_ESM.docx]

**Appendix**

**Mortality Implications of Increased Active Mobility for a Proposed Regional Transportation Emissions Cap-and-Invest Program**

**Authors:** Matthew Raifman, Kathy Fallon Lambert, Jonathan I. Levy, and Patrick L. Kinney

The Transportation Climate Initiative (TCI) made available to our research team nine GHG emissions and investment scenarios. In the main text, we presented our analysis of the potential physical activity benefits from the investment of proceeds of a 25% GHG emissions cap under three different investment scenarios (starred below). We conducted analyses on the three investment scenarios for two additional GHG emissions caps, 20% and 22%. As such, the full suite of scenarios we considered in our analysis were:

- Scenario 1: 25% GHG emissions cap; Scenario A investment scenario*
- Scenario 2: 25% GHG emissions cap; Scenario B investment scenario*
- Scenario 3: 25% GHG emissions cap; Scenario C investment scenario*
- Scenario 4: 22% GHG emissions cap; Scenario A investment scenario
- Scenario 5: 22% GHG emissions cap; Scenario B investment scenario
- Scenario 6: 22% GHG emissions cap; Scenario C investment scenario
- Scenario 7: 20% GHG emissions cap; Scenario A investment scenario
- Scenario 8: 20% GHG emissions cap; Scenario B investment scenario
- Scenario 9: 20% GHG emissions cap; Scenario C investment scenario

As an overview of the nine scenarios considered, below we present a complete breakdown of allocation of proceeds across the three investment scenarios (see Table 4), as well as summary of the net deaths avoided by state for the nine scenarios side-by-side (see Table 6). Recognizing the potential for a safety in numbers effect that would attenuate the traffic fatalities-to-exposure relationship with increased pedestrian and cycling volume, we also provide in Table 7 the estimated net deaths avoided for all nine scenarios assuming a safety-in-numbers coefficient of effect of 0.459 for cycling and 0.409 for walking, the most conservative estimate from a meta-analysis Elvik and Goal (2019). We then provide information on each specific scenario in the following sections of the Appendix.

To supplement output in the main text, where we focused on Scenario “B” output as a middle of the road allocation, we present in the appendix state-level deaths avoided, traffic fatalities, and monetary valuation (see Table 10). For the remainder of the scenarios, we share complete output in the appendix.

Note: totals in tables may differ from the sum of the data presented due to rounding for two significant figures.

**Table 4** Breakdown of allocation of TCI proceeds by investment strategy.

| **Investment Strategy** | **Scenario A** | **Scenario B** | **Scenario C** |
| --- | --- | --- | --- |
| **EV/alt fuel incentives** | **25.9%** | **52.7%** | **81.3%** |
| Light duty EV's | 4.6% | 30.0% | 54.0% |
| CNG trucks | 1.9% | 1.5% | 1.1% |
| Electric transit buses | 4.6% | 4.0% | 2.5% |
| Electric school buses | 4.6% | 5.9% | 7.1% |
| Electric trucks - MDT/urban | 4.6% | 9.0% | 15.0% |
| Hydrogen trucks - long-haul | 2.8% | 1.0% | 1.6% |
| Passenger rail electrification | 2.8% | 1.4% | 0.0% |
| **Vehicle travel reduction** | **16.2%** | **13.9%** | **10.5%** |
| Shared ride incentives | 1.9% | 0.9% | 0.0% |
| Land use/smart growth | 4.2% | 4.4% | 4.5% |
| Bicycle investment | 4.2% | 5.1% | 6.0% |
| Pedestrian investment | 4.2% | 3.0% | 0.0% |
| Travel demand management | 1.9% | 0.5% | 0.0% |
| **System efficiency** | **7.0%** | **7.6%** | **0.8%** |
| System operations | 1.9% | 1.1% | 0.4% |
| Freight/intermodal | 1.9% | 1.1% | 0.4% |
| Highway preservation | 3.3% | 5.4% | 0.0% |
| **Urban & intercity transit** | **24.2%** | **12.4%** | **0.0%** |
| Bus rapid transit | 2.8% | 1.4% | 0.0% |
| Urban rail | 2.8% | 1.4% | 0.0% |
| Commuter rail | 2.8% | 1.4% | 0.0% |
| Intercity rail | 2.8% | 1.4% | 0.0% |
| Bus service: expansion | 2.8% | 1.5% | 0.0% |
| Bus service: efficiency | 6.1% | 3.1% | 0.0% |
| Transit fare reduction | 4.2% | 2.3% | 0.0% |
| **Transit state of good repair** | **10.0%** | **5.0%** | **0.0%** |
| Bus | 3.3% | 1.7% | 0.0% |
| Urban rail | 3.3% | 1.7% | 0.0% |
| Commuter/intercity rail | 3.3% | 1.7% | 0.0% |
| **Indirect (non-GHG reducing)** | **16.7%** | **8.3%** | **7.4%** |
| **Total** | **100.0%** | **100.0%** | **100.0%** |

**Table 5** Annual TCI allocation to cycling and pedestrian infrastructure (millions USD).

|  | **25% GHG Emission Cap** | | | **22% GHG Emission Cap** | | | **20% GHG Emission Cap** | | |
| --- | --- | --- | --- | --- | --- | --- | --- | --- | --- |
| **State** | **Scenario A** | **Scenario B** | **Scenario C** | **Scenario A** | **Scenario B** | **Scenario C** | **Scenario A** | **Scenario B** | **Scenario C** |
| Cycling Investment Subset | $316 | $318 | $280 | $139 | $160 | $130 | $72 | $82 | $66 |
| Pedestrian Investment Subset | $316 | $188 | $0 | $139 | $96 | $0 | $72 | $48 | $0 |
| **Total Investments across all buckets** | **$7,600** | **$6,300** | **$4,600** | **$3,300** | **$3,200** | **$2,100** | **$1,700** | **$1,600** | **$1,100** |

**Table 6** Summary of annual net deaths avoided by state for the nine TCI scenarios considered without the safety-in-numbers effect.

|  | **25% GHG Emission Cap** | | | **22% GHG Emission Cap** | | | **20% GHG Emission Cap** | | |
| --- | --- | --- | --- | --- | --- | --- | --- | --- | --- |
| **State** | **Scenario A** | **Scenario B** | **Scenario C** | **Scenario A** | **Scenario B** | **Scenario C** | **Scenario A** | **Scenario B** | **Scenario C** |
| Connecticut | 8.9 | 7.4 | 5.4 | 3.9 | 3.7 | 2.6 | 2 | 1.9 | 1.3 |
| Delaware | 2.4 | 2.1 | 1.5 | 1 | 1 | 0.72 | 0.54 | 0.51 | 0.35 |
| D.C. | 17 | 14 | 9.7 | 9.9 | 10 | 6.6 | 5.1 | 5 | 3.3 |
| Maine | 1.7 | 1.2 | 0.63 | 0.64 | 0.47 | 0.23 | 0.33 | 0.24 | 0.11 |
| Maryland | 67 | 60 | 45 | 29 | 31 | 22 | 15 | 15 | 11 |
| Massachusetts | 28 | 24 | 17 | 14 | 14 | 9.7 | 7.3 | 7 | 4.6 |
| New Hampshire | 1.5 | 0.99 | 0.53 | 0.55 | 0.4 | 0.19 | 0.29 | 0.2 | 0.092 |
| New Jersey | 110 | 97 | 72 | 49 | 51 | 37 | 26 | 26 | 18 |
| New York | 360 | 280 | 170 | 200 | 190 | 120 | 100 | 96 | 57 |
| Pennsylvania | 71 | 58 | 40 | 38 | 37 | 24 | 19 | 18 | 12 |
| Rhode Island | 3.1 | 2.7 | 2 | 1.4 | 1.3 | 0.97 | 0.71 | 0.67 | 0.47 |
| Vermont | 0.65 | 0.46 | 0.25 | 0.24 | 0.18 | 0.092 | 0.13 | 0.092 | 0.044 |
| Virginia | 37 | 32 | 24 | 16 | 17 | 12 | 8.5 | 8.3 | 5.7 |
| **Total** | **710** | **580** | **390** | **360** | **360** | **240** | **190** | **180** | **110** |

**Table 7** Summary of annual net deaths avoided by state for the nine TCI scenarios considered with the safety-in-numbers effect.*

|  | **25% GHG Emission Cap** | | | **22% GHG Emission Cap** | | | **20% GHG Emission Cap** | | |
| --- | --- | --- | --- | --- | --- | --- | --- | --- | --- |
| **State** | **Scenario A** | **Scenario B** | **Scenario C** | **Scenario A** | **Scenario B** | **Scenario C** | **Scenario A** | **Scenario B** | **Scenario C** |
| Connecticut | 9.7 | 8.0 | 5.9 | 4.2 | 4.0 | 2.6 | 2.2 | 2.0 | 1.4 |
| Delaware | 2.6 | 2.2 | 1.6 | 1.1 | 1.1 | 0.72 | 0.57 | 0.54 | 0.37 |
| D.C. | 18 | 15 | 10 | 10 | 11 | 6.6 | 5.4 | 5.3 | 3.4 |
| Maine | 1.8 | 1.2 | 0.67 | 0.68 | 0.51 | 0.23 | 0.35 | 0.25 | 0.12 |
| Maryland | 70 | 63 | 47 | 30 | 32 | 22 | 16 | 16 | 11 |
| Massachusetts | 31 | 26 | 18 | 15 | 15 | 9.7 | 8 | 7.6 | 5.1 |
| New Hampshire | 1.6 | 1.1 | 0.57 | 0.6 | 0.44 | 0.19 | 0.31 | 0.22 | 0.1 |
| New Jersey | 120 | 110 | 78 | 53 | 56 | 37 | 28 | 28 | 19 |
| New York | 390 | 310 | 190 | 220 | 210 | 120 | 110 | 110 | 63 |
| Pennsylvania | 75 | 61 | 42 | 39 | 39 | 24 | 20 | 19 | 13 |
| Rhode Island | 3.4 | 2.9 | 2.1 | 1.5 | 1.5 | 0.97 | 0.76 | 0.73 | 0.51 |
| Vermont | 0.7 | 0.49 | 0.27 | 0.26 | 0.2 | 0.092 | 0.14 | 0.099 | 0.048 |
| Virginia | 41 | 35 | 26 | 18 | 18 | 12 | 9.3 | 9.1 | 6.2 |
| **Total** | **770** | **630** | **430** | **390** | **390** | **240** | **200** | **190** | **120** |

* applies a safety-in-numbers effect to the relationship between personal miles traveled and traffic fatalities from Elvik and Goal (2019) (0.459 for cycling and 0.409 for walking) to attenuate the relationship with increased volume from TCI investments.

**Detailed physical activity analysis for all five TCI scenario considered.**

Scenario 1: 25% GHG emissions cap with Scenario “A” investment allocation

**Table 8** Annual deaths avoided, traffic fatalities, net deaths, and monetary valuation by state for the 25% GHG emissions cap with the Scenario “A” allocation.

| **State** | **Deaths Avoided from Activity - Est.** | **Deaths Avoided from Activity – CI** | **Traffic Fatalities - Est.** | **Traffic Fatalities - CI** | **Net Deaths Avoided - Est.** | **Net Deaths Avoided - CI** | **Monetary Valuation - Est.** | **Monetary Valuation - CI** |
| --- | --- | --- | --- | --- | --- | --- | --- | --- |
| Connecticut | 10 | (6, 15) | 1.4 | (1.3, 1.4) | 8.9 | (5.8, 12) | $85 | ($36, $140) |
| Delaware | 2.7 | (1.6, 3.8) | 0.29 | (0.28, 0.31) | 2.4 | (1.6, 3.3) | $23 | ($9.8, $37) |
| District of Columbia | 18 | (10, 27) | 1.6 | (1.5, 1.7) | 17 | (11, 23) | $160 | ($68, $260) |
| Maine | 1.9 | (1, 2.8) | 0.2 | (0.19, 0.2) | 1.7 | (1, 2.4) | $16 | ($6.3, $26) |
| Maryland | 72 | (44, 100) | 5.2 | (5, 5.5) | 67 | (46, 88) | $640 | ($280, $1000) |
| Massachusetts | 33 | (19, 47) | 4.5 | (4.3, 4.7) | 28 | (19, 38) | $270 | ($110, $430) |
| New Hampshire | 1.7 | (0.87, 2.4) | 0.2 | (0.19, 0.21) | 1.5 | (0.88, 2) | $14 | ($5.4, $23) |
| New Jersey | 130 | (75, 180) | 16 | (15, 17) | 110 | (72, 150) | $1,000 | ($440, $1700) |
| New York | 420 | (230, 610) | 58 | (55, 60) | 360 | (220, 500) | $3,500 | ($1300, $5600) |
| Pennsylvania | 78 | (44, 110) | 6.9 | (6.6, 7.2) | 71 | (47, 95) | $680 | ($290, $1100) |
| Rhode Island | 3.6 | (2.1, 5) | 0.44 | (0.42, 0.46) | 3.1 | (2.1, 4.2) | $30 | ($13, $47) |
| Vermont | 0.74 | (0.4, 1.1) | 0.088 | (0.084, 0.092) | 0.65 | (0.4, 0.9) | $6.30 | ($2.5, $10) |
| Virginia | 44 | (26, 61) | 6.1 | (5.8, 6.4) | 37 | (25, 50) | $360 | ($150, $570) |
| **Total** | **810** | **(460, 1200)** | **100** | **(96, 110)** | **710** | **(450, 970)** | **$6800** | **($2700, $11000)** |

**Table 9** Annual change in annual physical activity and net deaths by county population density for the 25% GHG cap, Scenario “A” allocation.

| **County Type** | **Population Density per sq. mi.** | **Change in Bike PMT (millions)** | **Change in Walk PMT (millions)** | **Change in Bike Miles p.c.** | **Change in Walk Miles p.c.** | **Net Deaths Avoided** | **Net Deaths Avoided p.c.** |
| --- | --- | --- | --- | --- | --- | --- | --- |
| New York City | >70,000 | 220 | 150 | 160 | 87 | 170 | 14 |
| Urban Core | > 10,000- 70k | 250 | 84 | 31 | 8.4 | 190 | 2.7 |
| Urban | 4,000-10,000 | 380 | 47 | 94 | 9.2 | 210 | 5.8 |
| Suburban | 500-3,999 | 200 | 28 | 7.9 | 0.87 | 100 | 0.44 |
| Rural | <500 | 27 | 12 | 1.8 | 0.63 | 26 | 0.18 |
| **Total** |  | **1100** | **320** | **290** | **110** | **710** | **23** |

Scenario 2: 25% GHG emissions cap with Scenario “B” investment allocation

**Table 10** Annual deaths avoided, traffic fatalities, net deaths, and monetary valuation by state for the 25% GHG emissions cap with the Scenario “B” investment scenario.

| **State** | **Deaths Avoided from Activity - Est.** | **Deaths Avoided from Activity – CI** | **Traffic Fatalities - Est.** | **Traffic Fatalities – CI** | **Net Deaths Avoided - Est.** | **Net Deaths Avoided - CI** | **Monetary Valuation - Est.** | **Monetary Valuation - CI** |
| --- | --- | --- | --- | --- | --- | --- | --- | --- |
| Connecticut | 8.6 | (5.3, 12) | 1.2 | (1.1, 1.3) | 7.4 | (4.8, 10) | $71 | ($29, $110) |
| Delaware | 2.3 | (1.5, 3.2) | 0.26 | (0.24, 0.27) | 2.1 | (1.4, 2.8) | $20 | ($8.3, $31) |
| District of Columbia | 15 | (8.9, 21) | 1.3 | (1.3, 1.4) | 14 | (9.4, 18) | $130 | ($57, $210) |
| Maine | 1.3 | (0.76, 1.9) | 0.14 | (0.14, 0.15) | 1.2 | (0.78, 1.6) | $11 | ($4.8, $18) |
| Maryland | 65 | (41, 89) | 4.8 | (4.5, 5) | 60 | (40, 80) | $580 | ($250, $910) |
| Massachusetts | 27 | (17, 38) | 3.9 | (3.6, 4.1) | 24 | (15, 32) | $230 | ($94, $360) |
| New Hampshire | 1.1 | (0.66, 1.6) | 0.15 | (0.14, 0.15) | 0.99 | (0.66, 1.3) | $9.50 | ($4, $15) |
| New Jersey | 110 | (69, 150) | 15 | (14, 16) | 97 | (63, 130) | $930 | ($390, $1500) |
| New York | 330 | (190, 470) | 47 | (45, 49) | 280 | (180, 390) | $2,700 | ($1100, $4300) |
| Pennsylvania | 63 | (38, 89) | 5.8 | (5.5, 6.1) | 58 | (39, 76) | $550 | ($240, $870) |
| Rhode Island | 3 | (1.9, 4.2) | 0.39 | (0.36, 0.41) | 2.7 | (1.7, 3.6) | $25 | ($11, $40) |
| Vermont | 0.52 | (0.31, 0.74) | 0.066 | (0.063, 0.07) | 0.46 | (0.3, 0.61) | $4.40 | ($1.9, $6.9) |
| Virginia | 38 | (24, 52) | 5.4 | (5.1, 5.8) | 32 | (21, 44) | $310 | ($130, $500) |
| **Total** | **670** | **(400, 940)** | **85** | **(81, 89)** | **580** | **(380, 790)** | **$5600** | **($2300, $8900)** |

Scenario 3: 25% GHG emissions cap with Scenario “C” investment allocation.

**Table 11** Annual deaths avoided, traffic fatalities, net deaths, and monetary valuation by state for the 25% GHG emissions cap with the Scenario “C” allocation.

| **State** | **Deaths Avoided from Activity - Est.** | **Deaths Avoided from Activity - CI** | **Traffic Fatalities - Est.** | **Traffic Fatalities - CI** | **Net Deaths Avoided - Est.** | **Net Deaths Avoided - CI** | **Monetary Valuation - Est.** | **Monetary Valuation - CI** |
| --- | --- | --- | --- | --- | --- | --- | --- | --- |
| Connecticut | 6.3 | (4.1, 8.4) | 0.9 | (0.85, 0.96) | 5.4 | (3.2, 7.5) | $52 | ($20, $83) |
| Delaware | 1.7 | (1.1, 2.3) | 0.2 | (0.19, 0.21) | 1.5 | (0.93, 2.1) | $15 | ($5.7, $23) |
| District of Columbia | 11 | (7.1, 14) | 0.98 | (0.92, 1) | 9.7 | (6.1, 13) | $94 | ($37, $150) |
| Maine | 0.71 | (0.47, 0.95) | 0.085 | (0.08, 0.091) | 0.63 | (0.38, 0.87) | $6 | ($2.3, $9.7) |
| Maryland | 49 | (32, 66) | 3.7 | (3.4, 3.9) | 45 | (29, 62) | $440 | ($180, $700) |
| Massachusetts | 20 | (13, 27) | 2.9 | (2.7, 3.1) | 17 | (10, 24) | $160 | ($62, $260) |
| New Hampshire | 0.61 | (0.4, 0.82) | 0.086 | (0.08, 0.091) | 0.53 | (0.32, 0.74) | $5.10 | ($1.9, $8.2) |
| New Jersey | 83 | (55, 110) | 11 | (11, 12) | 72 | (43, 100) | $690 | ($260, $1100) |
| New York | 210 | (140, 280) | 31 | (29, 33) | 170 | (100, 250) | $1,700 | ($640, $2700) |
| Pennsylvania | 44 | (29, 60) | 4.3 | (4, 4.6) | 40 | (25, 55) | $380 | ($150, $620) |
| Rhode Island | 2.3 | (1.5, 3.1) | 0.3 | (0.28, 0.32) | 2 | (1.2, 2.8) | $19 | ($7.3, $31) |
| Vermont | 0.29 | (0.19, 0.39) | 0.04 | (0.038, 0.043) | 0.25 | (0.15, 0.35) | $2.40 | ($0.93, $3.9) |
| Virginia | 28 | (18, 37) | 4.2 | (3.9, 4.4) | 24 | (14, 33) | $230 | ($87, $370) |
| **Total** | **450** | **(300, 610)** | **60** | **(56, 64)** | **390** | **(240, 550)** | **$3800** | **($1500, $6100)** |

**Table 12** Annual change in annual physical activity and net deaths by county population density for the 25% GHG cap,

Scenario “C” allocation.

| **County Type** | **Population Density per sq. mi.** | **Change in Bike PMT (millions)** | **Change in Walk PMT (millions)** | **Change in Bike Miles p.c.** | **Change in Walk Miles p.c.** | **Net Deaths Avoided** | **Net Deaths Avoided p.c.** |
| --- | --- | --- | --- | --- | --- | --- | --- |
| New York City | >70,000 | 220 | 0 | 160 | 0 | 64 | 5.1 |
| Urban Core | > 10,000- 70k | 250 | 0 | 31 | 0 | 110 | 1.5 |
| Urban | 4,000-10,000 | 330 | 0 | 83 | 0 | 140 | 3.9 |
| Suburban | 500-3,999 | 180 | 0 | 6.9 | 0 | 66 | 0.28 |
| Rural | <500 | 22 | 0 | 1.4 | 0 | 9.9 | 0.069 |
| **Total** |  | **1000** | **0** | **280** | **0** | **390** | **11** |

Scenario 4: 22% GHG emissions cap with Scenario “A” investment scenario

**Table 13** Annual deaths avoided, traffic fatalities, net deaths, and monetary valuation by state for the 22% GHG emissions cap with the Scenario “A” investment scenario.

| **State** | **Deaths Avoided from Activity - Est.** | **Deaths Avoided from Activity – CI** | **Traffic Fatalities - Est.** | **Traffic Fatalities – CI** | **Net Deaths Avoided - Est.** | **Net Deaths Avoided - CI** | **Monetary Valuation - Est.** | **Monetary Valuation - CI** |
| --- | --- | --- | --- | --- | --- | --- | --- | --- |
| Connecticut | 4.5 | (2.6, 6.3) | 0.6 | (0.57, 0.63) | 3.9 | (2.5, 5.2) | $37 | ($15, $59) |
| Delaware | 1.2 | (0.68, 1.6) | 0.12 | (0.12, 0.13) | 1 | (0.67, 1.4) | $9.90 | ($4.1, $16) |
| District of Columbia | 11 | (6.4, 15) | 0.95 | (0.9, 1) | 9.9 | (6.8, 13) | $95 | ($41, $150) |
| Maine | 0.71 | (0.36, 1.1) | 0.072 | (0.07, 0.075) | 0.64 | (0.37, 0.92) | $6.20 | ($2.2, $10) |
| Maryland | 32 | (19, 44) | 2.3 | (2.2, 2.4) | 29 | (20, 39) | $280 | ($120, $440) |
| Massachusetts | 16 | (9.6, 23) | 2.3 | (2.1, 2.4) | 14 | (9.3, 19) | $140 | ($57, $210) |
| New Hampshire | 0.63 | (0.32, 0.94) | 0.074 | (0.072, 0.077) | 0.55 | (0.31, 0.8) | $5.30 | ($1.9, $8.7) |
| New Jersey | 57 | (34, 79) | 7.4 | (7, 7.7) | 49 | (33, 66) | $470 | ($200, $750) |
| New York | 230 | (130, 330) | 33 | (31, 34) | 200 | (130, 270) | $1,900 | ($780, $3000) |
| Pennsylvania | 41 | (24, 58) | 3.6 | (3.4, 3.7) | 38 | (25, 50) | $360 | ($150, $570) |
| Rhode Island | 1.6 | (0.93, 2.2) | 0.19 | (0.18, 0.2) | 1.4 | (0.91, 1.8) | $13 | ($5.5, $21) |
| Vermont | 0.28 | (0.14, 0.41) | 0.032 | (0.031, 0.033) | 0.24 | (0.14, 0.35) | $2.30 | ($0.86, $3.8) |
| Virginia | 19 | (11, 27) | 2.7 | (2.6, 2.8) | 16 | (11, 22) | $160 | ($66, $250) |
| **Total** | 420 | (240, 590) | 53 | (50, 55) | 360 | (240, 490) | $3,500 | ($1400, $5500) |

**Table 14** Annual change in annual physical activity and net deaths by county population density for the 22% GHG cap, Scenario “A” allocation.

| **County Type** | **Population Density per sq. mi.** | **Change in Bike PMT (millions)** | **Change in Walk PMT (millions)** | **Change in Bike Miles p.c.** | **Change in Walk Miles p.c.** | **Net Deaths Avoided** | **Net Deaths Avoided p.c.** |
| --- | --- | --- | --- | --- | --- | --- | --- |
| **New York City** | >70,000 | 180 | 66 | 130 | 38 | 100 | 8.1 |
| **Urban Core** | > 10,000- 70k | 170 | 37 | 22 | 3.7 | 110 | 1.6 |
| **Urban** | 4,000-10,000 | 170 | 20 | 42 | 4 | 93 | 2.5 |
| **Suburban** | 500-3,999 | 89 | 12 | 3.5 | 0.38 | 45 | 0.19 |
| **Rural** | <500 | 8.1 | 5.5 | 0.53 | 0.28 | 10 | 0.069 |
| **Total** |  | 620 | 140 | 200 | 46 | 360 | 12 |

Scenario 5: 22% GHG emissions cap with Scenario “B” investment scenario

**Table 15** Annual deaths avoided, traffic fatalities, net deaths, and monetary valuation by state for the 22% GHG emissions cap with the Scenario “B” allocation.

| **State** | **Deaths Avoided from Activity - Est.** | **Deaths Avoided from Activity - CI** | **Traffic Fatalities - Est.** | **Traffic Fatalities - CI** | **Net Deaths Avoided - Est.** | **Net Deaths Avoided - CI** | **Monetary Valuation - Est.** | **Monetary Valuation - CI** |
| --- | --- | --- | --- | --- | --- | --- | --- | --- |
| Connecticut | 4.3 | (2.7, 5.9) | 0.6 | (0.56, 0.63) | 3.7 | (2.4, 5) | $36 | ($15, $57) |
| Delaware | 1.1 | (0.71, 1.6) | 0.13 | (0.12, 0.13) | 1 | (0.66, 1.4) | $9.70 | ($4, $15) |
| District of Columbia | 11 | (6.7, 15) | 0.98 | (0.93, 1) | 10 | (6.8, 13) | $97 | ($42, $150) |
| Maine | 0.53 | (0.3, 0.76) | 0.057 | (0.054, 0.06) | 0.47 | (0.31, 0.64) | $4.50 | ($1.9, $7.2) |
| Maryland | 33 | (21, 45) | 2.4 | (2.3, 2.6) | 31 | (21, 41) | $300 | ($130, $460) |
| Massachusetts | 16 | (10, 23) | 2.3 | (2.2, 2.4) | 14 | (9.1, 19) | $130 | ($56, $210) |
| New Hampshire | 0.46 | (0.26, 0.67) | 0.058 | (0.055, 0.061) | 0.4 | (0.26, 0.55) | $3.90 | ($1.6, $6.2) |
| New Jersey | 59 | (37, 81) | 7.8 | (7.4, 8.3) | 51 | (33, 69) | $490 | ($200, $780) |
| New York | 230 | (130, 320) | 33 | (31, 34) | 190 | (130, 260) | $1,800 | ($770, $2900) |
| Pennsylvania | 41 | (25, 56) | 3.6 | (3.4, 3.8) | 37 | (25, 49) | $350 | ($150, $560) |
| Rhode Island | 1.5 | (0.97, 2.1) | 0.2 | (0.18, 0.21) | 1.3 | (0.88, 1.8) | $13 | ($5.4, $21) |
| Vermont | 0.21 | (0.12, 0.3) | 0.026 | (0.025, 0.027) | 0.18 | (0.12, 0.25) | $1.80 | ($0.73, $2.8) |
| Virginia | 19 | (12, 27) | 2.8 | (2.7, 3) | 17 | (11, 23) | $160 | ($65, $250) |
| **Total** | **410** | **(250, 580)** | **54** | **(51, 57)** | **360** | **(240, 480)** | **$3,400** | **($1400, $5500)** |

**Table 16** Annual change in annual physical activity and net deaths by county population density for the 22% GHG emissions cap with the Scenario “B” allocation.

| **County Type** | **Population Density per sq. mi.** | **Change in Bike PMT (millions)** | **Change in Walk PMT (millions)** | **Change in Bike Miles p.c.** | **Change in Walk Miles p.c.** | **Net Deaths Avoided** | **Net Deaths Avoided p.c.** |
| --- | --- | --- | --- | --- | --- | --- | --- |
| New York City | >70,000 | 210 | 44 | 150 | 25 | 94 | 7.6 |
| Urban Core | > 10,000- 70k | 200 | 24 | 25 | 2.5 | 110 | 1.6 |
| Urban | 4,000-10,000 | 200 | 14 | 49 | 2.7 | 99 | 2.7 |
| Suburban | 500-3,999 | 100 | 6.3 | 4.1 | 0.19 | 45 | 0.19 |
| Rural | <500 | 9.5 | 2.7 | 0.63 | 0.14 | 7.4 | 0.052 |
| **Total** |  | 720 | 91 | 230 | 31 | 360 | 12 |

Scenario 6: 22% GHG emissions cap with Scenario “C” investment scenario

**Table 17** Annual deaths avoided, traffic fatalities, net deaths, and monetary valuation by state for the 22% GHG emissions cap with the Scenario “C” allocation.

| **State** | **Deaths Avoided from Activity - Est.** | **Deaths Avoided from Activity - CI** | **Traffic Fatalities - Est.** | **Traffic Fatalities - CI** | **Net Deaths Avoided - Est.** | **Net Deaths Avoided - CI** | **Monetary Valuation - Est.** | **Monetary Valuation - CI** |
| --- | --- | --- | --- | --- | --- | --- | --- | --- |
| Connecticut | 2.8 | (1.9, 3.8) | 0.41 | (0.38, 0.43) | 2.4 | (1.5, 3.4) | $23 | ($8.9, $38) |
| Delaware | 0.76 | (0.5, 1) | 0.088 | (0.082, 0.094) | 0.67 | (0.41, 0.93) | $6.40 | ($2.5, $10) |
| District of Columbia | 6.8 | (4.5, 9.2) | 0.62 | (0.58, 0.66) | 6.2 | (3.9, 8.5) | $60 | ($24, $96) |
| Maine | 0.24 | (0.16, 0.32) | 0.029 | (0.027, 0.03) | 0.21 | (0.13, 0.29) | $2 | ($0.78, $3.2) |
| Maryland | 22 | (15, 30) | 1.7 | (1.6, 1.8) | 21 | (13, 28) | $200 | ($80, $320) |
| Massachusetts | 10 | (6.8, 14) | 1.5 | (1.4, 1.6) | 8.8 | (5.3, 12) | $85 | ($32, $140) |
| New Hampshire | 0.21 | (0.14, 0.28) | 0.029 | (0.027, 0.031) | 0.18 | (0.11, 0.25) | $1.70 | ($0.65, $2.7) |
| New Jersey | 39 | (26, 52) | 5.3 | (5, 5.7) | 34 | (20, 47) | $320 | ($120, $520) |
| New York | 130 | (85, 170) | 20 | (19, 21) | 110 | (65, 150) | $1,000 | ($400, $1700) |
| Pennsylvania | 25 | (16, 33) | 2.3 | (2.2, 2.5) | 23 | (14, 31) | $220 | ($86, $350) |
| Rhode Island | 1 | (0.68, 1.4) | 0.14 | (0.13, 0.14) | 0.9 | (0.55, 1.3) | $8.60 | ($3.3, $14) |
| Vermont | 0.098 | (0.065, 0.13) | 0.013 | (0.013, 0.014) | 0.084 | (0.051, 0.12) | $0.81 | ($0.31, $1.3) |
| Virginia | 13 | (8.4, 17) | 1.9 | (1.8, 2) | 11 | (6.5, 15) | $100 | ($40, $170) |
| **Total** | 250 | (170, 340) | 34 | (32, 36) | 220 | (130, 300) | $2100 | ($800, $3400) |

**Table 18** Annual change in annual physical activity and net deaths by county population density for the 22% GHG emissions cap with the Scenario “C” allocation.

| **County Type** | **Population Density per sq. mi.** | **Change in Bike PMT (millions)** | **Change in Walk PMT (millions)** | **Change in Bike Miles p.c.** | **Change in Walk Miles p.c.** | **Net Deaths Avoided** | **Net Deaths Avoided p.c.** |
| --- | --- | --- | --- | --- | --- | --- | --- |
| New York City | >70,000 | 160 | 0 | 120 | 0 | 48 | 3.9 |
| Urban Core | > 10,000- 70k | 160 | 0 | 20 | 0 | 69 | 0.96 |
| Urban | 4,000-10,000 | 150 | 0 | 38 | 0 | 66 | 1.8 |
| Suburban | 500-3,999 | 81 | 0 | 3.2 | 0 | 30 | 0.13 |
| Rural | <500 | 7.4 | 0 | 0.49 | 0 | 3.3 | 0.023 |
| **Total** |  | **560** | **0** | 180 | **0** | 220 | 6.8 |

Scenario 7: 20% GHG emissions cap with Scenario “A” investment allocation

**Table 19** Annual deaths avoided, traffic fatalities, net deaths, and monetary valuation by state for the 20% GHG emissions cap with the Scenario “A” allocation.

| **State** | **Deaths Avoided from Activity - Est.** | **Deaths Avoided from Activity - CI** | **Traffic Fatalities - Est.** | **Traffic Fatalities - CI** | **Net Deaths Avoided - Est.** | **Net Deaths Avoided - CI** | **Monetary Valuation - Est.** | **Monetary Valuation – CI** |
| --- | --- | --- | --- | --- | --- | --- | --- | --- |
| Connecticut | 2.3 | (1.4, 3.3) | 0.31 | (0.29, 0.32) | 2 | (1.3, 2.7) | $19 | ($8, $30) |
| Delaware | 0.6 | (0.35, 0.85) | 0.065 | (0.061, 0.068) | 0.54 | (0.35, 0.72) | $5.10 | ($2.1, $8.2) |
| District of Columbia | 5.6 | (3.3, 8) | 0.49 | (0.47, 0.52) | 5.1 | (3.5, 6.8) | $49 | ($21, $78) |
| Maine | 0.37 | (0.19, 0.55) | 0.037 | (0.036, 0.039) | 0.33 | (0.19, 0.47) | $3.20 | ($1.2, $5.2) |
| Maryland | 16 | (9.9, 23) | 1.2 | (1.1, 1.2) | 15 | (10, 20) | $150 | ($63, $230) |
| Massachusetts | 8.5 | (5, 12) | 1.2 | (1.1, 1.2) | 7.3 | (4.8, 9.8) | $70 | ($29, $110) |
| New Hampshire | 0.33 | (0.16, 0.49) | 0.038 | (0.037, 0.04) | 0.29 | (0.16, 0.41) | $2.80 | ($0.98, $4.5) |
| New Jersey | 29 | (18, 41) | 3.8 | (3.6, 4) | 26 | (17, 34) | $250 | ($100, $390) |
| New York | 120 | -68,170 | 17 | (16, 18) | 100 | -66,140 | $990 | ($400, $1600) |
| Pennsylvania | 21 | (12, 30) | 1.8 | (1.8, 1.9) | 19 | (13, 26) | $190 | ($79, $290) |
| Rhode Island | 0.81 | (0.48, 1.1) | 0.099 | (0.094, 0.1) | 0.71 | (0.47, 0.95) | $6.80 | ($2.9, $11) |
| Vermont | 0.14 | (0.073, 0.21) | 0.017 | (0.016, 0.017) | 0.13 | (0.073, 0.18) | $1.20 | ($0.45, $2) |
| Virginia | 9.9 | (5.9, 14) | 1.4 | (1.3, 1.5) | 8.5 | (5.6, 11) | $82 | ($34, $130) |
| **Total** | 220 | -120,310 | 27 | (26, 29) | 190 | (120, 250 | $1,800 | ($750, $2900) |

**Table 20** Annual change in annual physical activity and net deaths by county population density for the 20% GHG emissions cap with the Scenario “A” allocation.

| **County Type** | **Population Density per sq. mi.** | **Change in Bike PMT (millions)** | **Change in Walk PMT (millions)** | **Change in Bike Miles p.c.** | **Change in Walk Miles p.c.** | **Net Deaths Avoided** | **Net Deaths Avoided p.c.** |
| --- | --- | --- | --- | --- | --- | --- | --- |
| New York City | >70,000 | 92 | 34 | 66 | 20 | 52 | 4.2 |
| Urban Core | >10,000-70k | 90 | 19 | 11 | 1.9 | 59 | 0.82 |
| Urban | 4,000-10,000 | 87 | 11 | 22 | 2.1 | 48 | 1.3 |
| Suburban | 500-3,999 | 46 | 6.5 | 1.8 | 0.2 | 23 | 0.099 |
| Rural | <500 | 4.2 | 2.8 | 0.28 | 0.14 | 5.2 | 0.036 |
| **Total** |  | 320 | 73 | 100 | 24 | 190 | 6.5 |

Scenario 8: 20% GHG emissions cap with Scenario “B” investment allocation

**Table 21** Annual deaths avoided, traffic fatalities, net deaths, and monetary valuation by state for the 20% GHG emissions cap with the Scenario “B” allocation.

| **State** | **Deaths Avoided from Activity - Est.** | **Deaths Avoided from Activity - CI** | **Traffic Fatalities - Est.** | **Traffic Fatalities - CI** | **Net Deaths Avoided - Est.** | **Net Deaths Avoided - CI** | **Monetary Valuation - Est.** | **Monetary Valuation – CI** |
| --- | --- | --- | --- | --- | --- | --- | --- | --- |
| Connecticut | 2.2 | (1.3, 3) | 0.3 | (0.28, 0.32) | 1.9 | (1.2, 2.5) | $18 | ($7.3, $28) |
| Delaware | 0.57 | (0.36, 0.78) | 0.064 | (0.06, 0.067) | 0.51 | (0.33, 0.68) | $4.90 | ($2, $7.7) |
| District of Columbia | 5.5 | (3.4, 7.7) | 0.49 | (0.46, 0.52) | 5 | (3.4, 6.7) | $48 | ($21, $76) |
| Maine | 0.27 | (0.15, 0.38) | 0.029 | (0.027, 0.03) | 0.24 | (0.15, 0.32) | $2.30 | ($0.94, $3.6) |
| Maryland | 17 | (10, 23) | 1.2 | (1.1, 1.3) | 15 | (10, 20) | $150 | ($63, $230) |
| Massachusetts | 8.1 | (5, 11) | 1.2 | (1.1, 1.2) | 7 | (4.6, 9.4) | $67 | ($28, $110) |
| New Hampshire | 0.23 | (0.13, 0.33) | 0.029 | (0.028, 0.03) | 0.2 | (0.13, 0.27) | $1.90 | ($0.79, $3.1) |
| New Jersey | 30 | (18, 41) | 3.9 | (3.7, 4.1) | 26 | (17, 35) | $250 | ($100, $390) |
| New York | 110 | (67, 160) | 16 | (16, 17) | 96 | (63, 130) | $920 | ($380, $1500) |
| Pennsylvania | 20 | (12, 28) | 1.8 | (1.7, 1.9) | 18 | (12, 24) | $180 | ($76, $280) |
| Rhode Island | 0.77 | (0.48, 1.1) | 0.098 | (0.092, 0.1) | 0.67 | (0.44, 0.91) | $6.50 | ($2.7, $10) |
| Vermont | 0.11 | (0.059, 0.15) | 0.013 | (0.012, 0.014) | 0.092 | (0.06, 0.12) | $0.89 | ($0.37, $1.4) |
| Virginia | 9.7 | (6, 13) | 1.4 | (1.3, 1.5) | 8.3 | (5.4, 11) | $80 | ($33, $130) |
| **Total** | **210** | **(130, 290)** | **27** | **(25, 28)** | **180** | **(120, 240)** | **$1700** | **($720, $2700)** |

**Table 22** Annual change in annual physical activity and net deaths by county population density for the 20% GHG emissions cap with the Scenario “B” allocation.

| **County Type** | **Population Density per sq. mi.** | **Change in Bike PMT (millions)** | **Change in Walk PMT (millions)** | **Change in Bike Miles p.c.** | **Change in Walk Miles p.c.** | **Net Deaths Avoided** | **Net Deaths Avoided p.c.** |
| --- | --- | --- | --- | --- | --- | --- | --- |
| New York City | >70,000 | 100 | 22 | 75 | 13 | 47 | 3.8 |
| Urban Core | >10,000-70k | 100 | 12 | 13 | 1.2 | 57 | 0.79 |
| Urban | 4,000-10,000 | 98 | 6.9 | 24 | 1.3 | 49 | 1.3 |
| Suburban | 500-3,999 | 52 | 3.2 | 2 | 0.097 | 22 | 0.096 |
| Rural | <500 | 4.8 | 1.4 | 0.31 | 0.068 | 3.7 | 0.026 |
| **Total** |  | **360** | **46** | **110** | **15** | **180** | **6.0** |

Scenario 9: 20% GHG emissions cap with Scenario “C” investment allocation

**Table 23** Annual deaths avoided, traffic fatalities, net deaths, and monetary valuation by state for the 20% GHG emissions cap with the Scenario “C” allocation.

| **State** | **Deaths Avoided from Activity - Est.** | **Deaths Avoided from Activity - CI** | **Traffic Fatalities - Est.** | **Traffic Fatalities - CI** | **Net Deaths Avoided - Est.** | **Net Deaths Avoided - CI** | **Monetary Valuation - Est.** | **Monetary Valuation – CI** |
| --- | --- | --- | --- | --- | --- | --- | --- | --- |
| Connecticut | 1.5 | (0.97, 2) | 0.21 | (0.2, 0.23) | 1.3 | (0.76, 1.8) | $12 | ($4.6, $20) |
| Delaware | 0.4 | (0.26, 0.53) | 0.046 | (0.043, 0.049) | 0.35 | (0.21, 0.48) | $3.40 | ($1.3, $5.4) |
| District of Columbia | 3.6 | (2.4, 4.8) | 0.33 | (0.31, 0.35) | 3.3 | (2, 4.5) | $31 | ($12, $50) |
| Maine | 0.12 | (0.082, 0.17) | 0.015 | (0.014, 0.016) | 0.11 | (0.067, 0.15) | $1.10 | ($0.41, $1.7) |
| Maryland | 12 | (7.7, 16) | 0.87 | (0.81, 0.93) | 11 | (6.8, 15) | $100 | ($42, $170) |
| Massachusetts | 5.4 | (3.6, 7.3) | 0.8 | (0.75, 0.86) | 4.6 | (2.8, 6.5) | $44 | ($17, $72) |
| New Hampshire | 0.11 | (0.071, 0.14) | 0.015 | (0.014, 0.016) | 0.092 | (0.056, 0.13) | $0.89 | ($0.34, $1.4) |
| New Jersey | 20 | (13, 27) | 2.8 | (2.6, 3) | 18 | (11, 25) | $170 | ($65, $270) |
| New York | 68 | (44, 91) | 10 | (9.7, 11) | 57 | (34, 80) | $550 | ($210, $890) |
| Pennsylvania | 13 | (8.6, 18) | 1.2 | (1.1, 1.3) | 12 | (7.4, 16) | $110 | ($45, $180) |
| Rhode Island | 0.54 | (0.36, 0.73) | 0.071 | (0.067, 0.076) | 0.47 | (0.29, 0.66) | $4.50 | ($1.7, $7.3) |
| Vermont | 0.051 | (0.034, 0.069) | 0.007 | (0.0066, 0.0075) | 0.044 | (0.027, 0.062) | $0.42 | ($0.16, $0.69) |
| Virginia | 6.7 | (4.4, 9) | 1 | (0.94, 1.1) | 5.7 | (3.4, 8) | $55 | ($21, $88) |
| **Total** | 130 | (86, 180) | 18 | (17, 19) | 110 | (69, 160) | $1,100 | ($420, $1800) |

**Table 24** Annual change in annual physical activity and net deaths by county population density for the 20% GHG emissions cap with the Scenario “C” allocation.

| **County Type** | **Population Density per sq. mi.** | **Change in Bike PMT (millions)** | **Change in Walk PMT (millions)** | **Change in Bike Miles p.c.** | **Change in Walk Miles p.c.** | **Net Deaths Avoided** | **Net Deaths Avoided p.c.** |
| --- | --- | --- | --- | --- | --- | --- | --- |
| New York City | >70,000 | 84 | 0 | 61 | 0 | 25 | 2 |
| Urban Core | >10,000-70k | 83 | 0 | 10 | 0 | 36 | 0.5 |
| Urban | 4,000-10,000 | 80 | 0 | 20 | 0 | 35 | 0.94 |
| Suburban | 500-3,999 | 42 | 0 | 1.7 | 0 | 16 | 0.067 |
| Rural | <500 | 3.9 | 0 | 0.25 | 0 | 1.7 | 0.012 |
| **Total** |  | 290 | 0 | 93 | 0 | 110 | 3.5 |

**Table 25** Baseline mortality rate per 100,000 by county population density.

| **County Type** | **Population Density per sq. mi.** | **Age 20-64** | **Age 20-74** |
| --- | --- | --- | --- |
| New York City | >70,000 | 241 | 366 |
| Urban Core | >10,000-70k | 329 | 474 |
| Urban | 4,000-10,000 | 304 | 445 |
| Suburban | 500-3,999 | 345 | 536 |
| Rural | <500 | 375 | 595 |
| Total |  | 364 | 573 |
